# Impact of an Educational Program on Behavioral Changes toward Environmental Health among Laotian Students

**DOI:** 10.3390/ijerph17145055

**Published:** 2020-07-14

**Authors:** Jiyoung Shin, Harris Hyun-soo Kim, Eun Mee Kim, Yookyung Choi, Eunhee Ha

**Affiliations:** 1 Department of Occupational and Environmental Medicine, College of Medicine, Ewha Womans University, Seoul 07804, Korea; nellshin5@gmail.com; 2 Department of Sociology, College of Social Sciences, Ewha Womans University, Seoul 03760, Korea; harrishkim@ewha.ac.kr; 3 Department of International Studies, Graduate School of International Studies, Ewha Womans University, Seoul 03760, Korea; emkim@ewha.ac.kr; 4 Division of Kinesiology & Sports Studies, College of Science and Industry Convergence, Ewha Womans University, Seoul 03760, Korea; ykchoi0216@ewha.ac.kr

**Keywords:** Lao people’s democratic republic, school-based intervention, risk perception, gender, girls, environmental health

## Abstract

This study evaluates the effect of an integrated health care educational program on several behavioral changes related to environmental health among Laotian students. Students in the experimental group received education concerning environmental health-related issues, including air pollution and chemical exposure. Analyses of covariance (ANCOVA) and paired *t*-tests were conducted for the statistical analysis of the pre- and post-survey scores. The post-test scores of the experimental group regarding their risk perception and information-seeking behaviors towards air pollution and chemical exposure were higher than those of the control group after controlling for the pre-test scores. Moreover, in the experimental group, the girls’ risk perception scores significantly increased after receiving the education, which was not observed in the control group. The risk perception score among non-drinking students also significantly increased after the program. These results indicate that the education program effectively enhanced the students’ risk perception, especially that of girls and nondelinquent students.

## 1. Introduction

Air pollution accounts for the greatest burden of disease worldwide [1]. Health risks related to indoor and outdoor air pollution are likely to be greatest in cities in developing countries, where risks are especially associated with solid fuel combustion and gasoline consumption [2]. Lao People’s Democratic Republic (Lao PDR, Laos) has witnessed an improvement in economic indexes, such as trade cooperation and productivity related to manufacturing expansion and industrialization. However, steady economic growth and a rapid increase in the number of vehicles have contributed to air pollution in Laos [3]. In addition, as most households in the country use wood stoves, emissions from wood burning are a major source of indoor air pollution [4].

Laos has many sources of chemical exposure, including pesticides, industrial by-products, chemicals used in plastics, and persistent organic pollutants. These substances are endocrine-disrupting chemicals, which are compounds that mimic or interfere with the normal actions of the endocrine hormones in the human body. Daily exposure to multiple chemicals may help increase the prevalence of asthma, autism, cancer, and other illnesses [5,6]. In Laos, farmers are highly likely to become pesticide-dependent, as several neighboring countries produce pesticides that are readily available. Many pesticides that have been banned in many developed countries, such as methyl parathion and monocrotophos, are still actively sold in local markets in Laos [7].

Exposure to air pollutants and chemicals is likely to have more adverse effects on children and adolescents than adults [8]. Children are most vulnerable to the harmful effects of ambient air pollutants because their defense mechanisms are still evolving and they inhale a higher volume of air per body weight than adults [9]. Moreover, adolescents’ exposure to chemical and biological risks in their environments including home, school, and workplace deserves attention and needs to be recognized as an important threat to their development. However, adolescence is underrepresented in the literature despite the fact that many organs and systems undergo significant development during this period [10].

In 2015, all 193 United Nations (UN) member states adopted Sustainable Development Goals (SDGs) to achieve economic, social, and environmental development, emphasizing that the world must work to combat poverty and improve the health of girls, women, and children from 2016 to 2030. SDG goal 3 is “Ensure healthy lives and promote well-being for all at all ages”, and goal 5 is, “Achieve gender equality and empower all women and girls.” This is the first time that adolescents and in particular, girls, were highlighted in the UN goals. Targets include special attention to girls’ health and rights, and thus, we have reviewed Laos’ situation with a focus on the health of girls and adolescents. Lao PDR has one of the highest adolescent pregnancy rates in Asia, and there are many inequalities in Laotian girls’ health opportunities and outcomes. Lao PDR also has a young population, with 60% of its over 6 million inhabitants estimated to be under 25 years of age. Investment in Laotian child and adolescent capital development, particularly in the areas of education, health, and participation in decision making, will ensure that every young person’s potential is fulfilled [11].

Therefore, it is important to develop multidisciplinary educational programs in Laos to raise the awareness of adolescents, especially girls, about the health risks they face at school and at home and to identify ways to make their environments safer [12]. Such programs should aim to enhance teenagers’ environmental risk knowledge, attitudes, and behaviors to equip young people with what they need to analyze and address in terms of environmental risks [13]. However, there are only a few studies regarding environmental health programs for children and adolescents in Laos.

Therefore, in this study, we implemented an educational program for adolescent students, especially girls, in Laos on environmental threats, namely, air pollution and chemical exposure. We then examined their environmental health-related behaviors before and after the program. 

## 2. Methods

### 2.1. Study Population

This study was a part of the Training Girls’ Integrated Health Care Specialists and Strengthening Girls’ Health Empowerment in Laos Project of Ewha Womans University, which was funded by the Korea International Cooperation Agency (KOICA) for the period 2017–2019. The project aimed to strengthen girls’ integrated health care specialists and girls’ empowerment by training Laotian specialists and educating students. The education program was implemented from 25 September to 23 November 2019. Six upper secondary schools in Vientiane, the capital, were randomly selected using a random number table. The project researchers asked the selected school principals whether their schools would like to participate in the project. The schools whose principals agreed to participate were included in our study. Four of these schools were selected for the experimental group, and two others were assigned to the control group. A total of 1065 5th-grade and 6th-grade students in the four schools in the experimental group (Saysetha, Chao Anouvong, Chansavang, Phiavat) participated in the Girls’ Integrated Health Care Program. The control group had 210 5th-grade and 6th-grade students (Nongbone, Nongduang). The selected students and their parents were asked for their consent to participate in the study, and the research was conducted for the students who agreed. Consent was obtained from 400 students and their parents from the experimental group, and 210 students from the control group.

### 2.2. Study Curriculum

The Girls’ Integrated Health Care Program was divided into two activities, namely, Girls’ Integrated Health Care Education and Peer Girls’ Special Group Activity. The Girls’ Integrated Health Care Education consisted of seven health-related subjects, namely, health behavior, social relationships, environmental health, nutrition, physical activity, gender and sexuality, and sexual and reproductive health. These seven subjects were selected from a focus group discussion of the Girls’ Integrated Health Care research team at Ewha Womans University. The research team used Langer et al.’s study on women’s health to identify integrated and comprehensive determinants of health with a focus on girls and women; sexual reproductive health and rights as well as gender and sexuality were regarded as important but neglected dimensions of health [14]. The curriculum, which was developed based on this comprehensive understanding of health, consisted of 24 sessions held over eight weeks; three of the 24 sessions (over two weeks) focused on environmental health. We developed an environmental health education textbook regarding air pollution and chemical use based on educational materials from the Korean Environmental Education Portal and the World Health Organization (WHO)’s training modules to address a range of environmental and health issues that have an impact on children [15,16]. Our education textbook regarding environmental health includes a series of lessons titled Indoor/outdoor Air Pollution, Sick Building Syndrome, Endocrine System, Endocrine Disruptors, Pesticide Use, and Clean House and Classroom. The education textbook was in flip chart format, which is easily accessed by schools. 

The program also included activities such as games and quizzes about air pollution and chemical use to make the students understand hazardous air pollution and chemical exposure easily and teach them how to keep their homes and classrooms clean from these exposures. The textbook and educational materials were developed in English and then translated into Lao. They were edited under the supervision of the Ministry of Education and Sports of Lao PDR. 

The 400 students in the experimental group received the Girls’ Integrated Health Care Education for eight weeks. Lao teachers from each experimental school developed the educational materials together with the research team to encourage the students to participate in the program, and these educational materials were used to teach the students.

Given that the purpose of this activity was to strengthen girls’ empowerment by enhancing their ability to solve problems themselves through group activities, 100 girls from the experimental group participated in the Peer Girls’ Special Group Activity in the same period. This activity, which was based on jumping rope, also contained seven health-related practices and an in-depth discussion. Two sessions of this activity were conducted per week, for a total of 16 sessions over eight weeks. One session’s discussion topic during one week was environmental pollution.

The 210 students in the control group did not participate in the education program and activities during the same period. Tests were administered before and after the intervention. Participants who could not complete the test were excluded; a total of 578 students (370 from the experimental group and 208 from the control group) were finally included in this study.

### 2.3. Ethics Approval

The study was approved by the Institutional Review Board of Ewha Womans University, Seoul, Korea (IRB number: 165-16).

### 2.4. Questionnaire and Outcome Measures

In many health education theories and models, risk perception, self-efficacy, and information seeking are key constructs required to change health behaviors [17,18]. Perceived risk is the belief that one is vulnerable to a disease or risk factor, and it is a significant predictor of self-protective behaviors [19]. Self-efficacy is one’s belief in his/her ability to achieve behavioral changes in specific situations [20]. These perceptions contribute to an individual’s judgments of his/her abilities to perform a specific behavior and hugely influence his/her choice and maintenance of that behavior. In this study, we evaluated students’ self-efficacy in obtaining information related to air pollution and chemical exposure. 

Information-seeking behavior is an important element of dealing effectively with uncertainty and risky situations [21,22]. Health-threatening environmental problems, such as air pollution, may cause people to experience strong negative emotions and motivate them to learn more about these issues in order to engage in preventive behaviors [23]. However, information avoidance could occur when individuals feel that there is too large a gap between what they know and what they need to know or when they feel they cannot do anything to mitigate the situation [24]. Therefore, determining whether the study participants actually engaged in information seeking about air pollution and chemical exposure after the educational program was important. 

To measure these health behaviors in relation to air pollution and chemical exposure, we developed a questionnaire. Considering different measurements in the literature regarding these behaviors, we produced 10 items to measure the students’ behavioral changes [25,26,27] (Table 1). Of these, four items were designed to assess risk perception, three to assess self-efficacy, and three to assess information-seeking behavior. Each item was rated on a seven-point Likert scale ranging from *Not at all* to *Extremely*, giving a possible score between 1 and 7 for each item. Thus, the possible total risk perception score ranged between 4 and 28, while the total scores for self-efficacy and information seeking ranged between 3 and 21. A greater score difference between the pre- and post-tests indicates greater behavioral change. The students were instructed to choose only one option per question. The internal consistency of the instruments (risk perception, self-efficacy, information seeking) was assessed using Cronbach’s alpha coefficient, with values equal to or greater than 0.70 considered satisfactory [28]. The values of all pre- and post-tests were greater than 0.70 (Table S1).

### 2.5. Statistical Analysis

Demographic characteristics of the experimental and control groups were compared using $\chi^{2}$ tests for categorical variables and *t*-tests for continuous variables for the statistical analysis. To analyze data with a continuous dependent variable, between-subjects predictor variable, and a continuous covariate, we conducted analyses of covariance (ANCOVAs) with each group (experimental, control) as the between-subjects factor to examine immediate pre-to-post-intervention effects. The group was used as a fixed factor, and the pre-test score, gender, school grade, smoking habit, and alcohol habit were regarded as confounders. We selected drinking habit and smoking habit as covariates to assess the students’ delinquent behavior. In the questionnaire, the students were asked how often they smoked and drank alcohol. Students who answered “never” were categorized as never drinkers/smokers. Students who answered “more than once or twice a year” were considered as having drinking/smoking habits and categorized as ever drinkers/smokers. 

Changes in the scores were analyzed within groups by paired *t*-tests stratified by the students’ gender and drinking and smoking habits, given that gender and juvenile delinquency have been found to modify the effect of education in previous studies [29,30,31]. A total of 367 pre-tests and post-tests in the experimental group and 206 in the control group were matched for analysis. 

To assess the effect of Peer Girls’ Special Group Activity on girls, we compared the post-test scores between them after different educational activities. One group consisted of girl students who participated in Girls’ Integrated Health Care Education only, and another group consisted of girl students who participated in both the Girls’ Integrated Health Care Education and Peer Girls’ Special Group Activity. All *p*-values were two-sided, and those less than 0.05 were considered significant. The statistical analyses were performed using the SAS 9.4 (SAS Institute, Cary, NC, USA). 

## 3. Results

### 3.1. Baseline Demographics

Of the 578 participants, 45.2% were 5th-graders, 40.7% were boys, and 4.3% smoked cigarettes more than once or twice a year. Among all the participants, 370 students (64%) were assigned to the experimental group. In this group, 56.2% were girls. In the control group, 64.9% of the participants were girls. The distribution of age, body mass index (BMI), and drinking habits did not differ substantially between the two groups (Table 2).

### 3.2. Risk Perception

Table 3 compares the behavioral post-test scores of the two groups after controlling for the covariates. ANCOVA results showed a significant difference between the risk perception scores (sum of the scores for the four risk perception variables) of the two groups after the intervention (Control group M = 22.57, SD = 3.25) (Experimental group M = 23.38, SD = 3.68) after adjusting for the baseline pre-test scores, gender, school grade, drinking habits, and smoking habits.

In Table S2, we compared the post-test scores between the girls in the experimental group to estimate the discussion activity’s effect and found no significant difference in the health behavior scores of the two groups (Education only group vs. Education + special activity group).

### 3.3. Self-efficacy

In Table 3, the ANCOVA results regarding self-efficacy (sum of the scores for the three self-efficacy variables) did not reveal statistically significant differences between the groups’ post-test scores after adjusting for the covariates (Control group M = 12.80, SD = 3.24) (Experimental group M = 13.87, SD = 3.23). A comparison of the post-test scores between the two activity groups among the girls showed no significant difference in self-efficacy scores (Education-only group M = 24.02, SD = 3.28) (Education + special activity group M = 24.12, SD = 3.33).

### 3.4. Information Seeking

Our comparison of the post-test scores between the groups revealed that the score of information-seeking behavior (sum of the scores for the three information-seeking variables) significantly increased in the experimental group after the program (Control group M = 12.74, SD = 3.21) (Experimental group M = 13.83, SD = 3.42). When comparing the different activities’ groups in girls, the information-seeking scores of the two groups were not significantly different (Education-only group M = 14.50, SD = 3.09) (Education + special activity group M = 14.42, SD = 3.02).

### 3.5. Stratification Analysis

**Figure 1:**
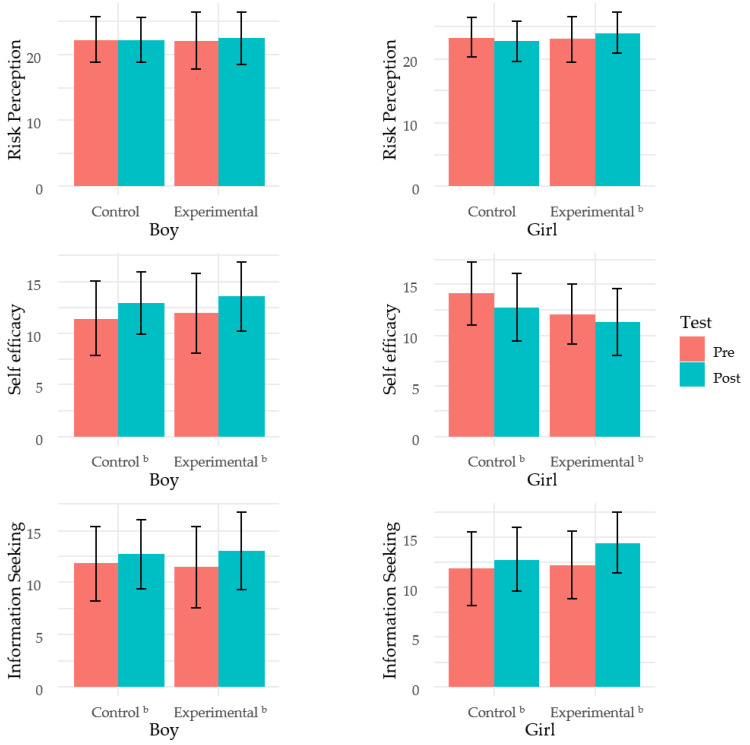
Comparison between pre- and post-test scores stratified by gender. ^a^ The *p*-values represent the score differences between the pre- and post-tests. ^b^ *p* < 0.05.

We also examined the program’s effect stratified by the students’ gender and delinquent behaviors. We found that risk perception significantly increased after the program among the girls in the experimental group, whereas no significant increase was observed among the girls in the control group (Figure 1).

**Figure 2:**
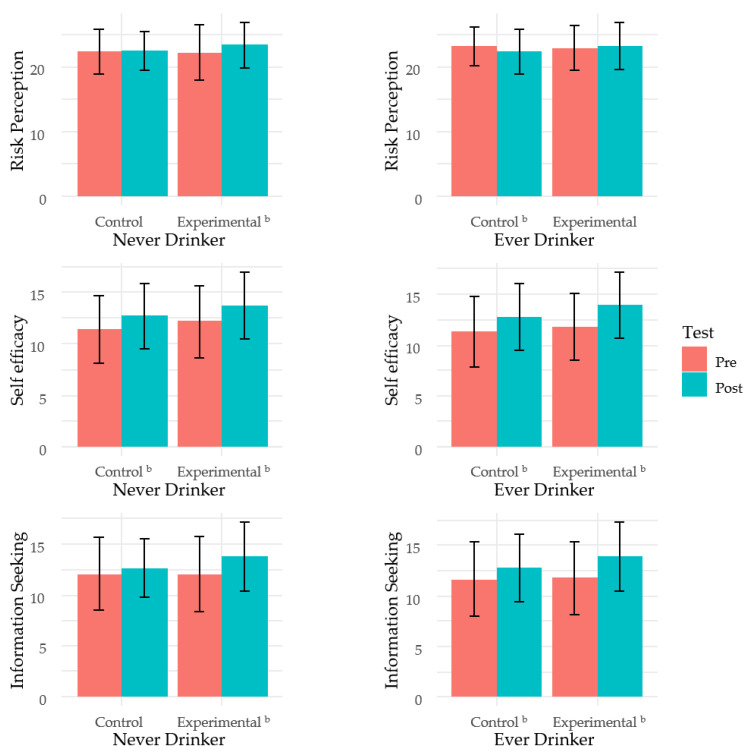
Comparison between pre- and post-test scores stratified by drinking habits. ^a^ The *p*-values represent the score differences between the pre- and post-tests. ^b^ *p* < 0.05

An examination of the results stratified by drinking habits showed that the risk perception and information-seeking scores of the non-drinking students in the experimental group significantly increased after the education, whereas those of the non-drinking students in the control group did not increase significantly. Among the drinking students, there was a significant increase in the self-efficacy and information-seeking scores after the education. However, a significant increase in these two variables was also observed in the control group. The risk perception score of the drinking students in the control group significantly decreased after the education (Figure 2).

Stratification of the results by smoking habits revealed that the risk perception scores of the never-smokers significantly increased after the education. No significant difference in risk perception scores was observed among the never-smokers in the control group (Table S3).

## 4. Discussion

To our knowledge, this study is the first to assess the effect of an environmental health care education program on health-related behaviors (risk perception, self-efficacy, and information seeking) among Laotian students. Our study found that the risk perception and information-seeking behavior scores of the students increased after the educational program.

It is important to develop such health education programs in Laos, especially among girls. Despite the country’s steady economic growth over the last two decades, meaningful development has not been sufficient to adequately decrease the persistent health and education inequity across the country, particularly among women [32,33]. Gough also argued that gender should be prioritized by environmental education researchers and journals to better achieve gender equality and more fully address the environmental emergency within the field [34].

The health-related behavior measurements regarding air pollution and chemical exposure developed by this study obtained good validity scores. In boys, the self-efficacy scores were higher in the post-test compared with those in the pre-test in both the experimental and control groups. On the other hand, girls’ self-efficacy scores were lower in the post-test compared with those in the pre-test in both groups. This score change could be the effect of traditional education in the groups during the study period. In other words, the change in the self-efficacy scores in the experimental group might not be considered an effect of our special education.

The stratification analysis also revealed significant improvements in risk perception after the education, especially among girls. Several studies have found that gender significantly influences environmental risk perception and suggest that males exhibit lower risk perceptions compared with women [35,36]. Therefore, this gender effect may have influenced the boys’ risk perception score in our study. This effect difference might be related to the Peer Girls’ Special Group Activity program, which was provided to some of the girl students only. Although there was no significant increase in the health behavior scores of the girls after this additional program, this activity contained a discussion regarding how to reduce environmental pollution. This in-depth discussion might have helped increase the girls’ risk perception regarding air pollution and chemical exposure compared with that of the boys. 

A significant improvement in risk perception was observed only among non-drinking and non-smoking students after the education. This might indicate that environmental health programs targeting students exhibiting delinquent behavior to improve their risk perception need to implement improved approaches to education.

This study has several strengths. Firstly, it uses a quasi-experimental study design with a comparison group. We targeted randomly selected Laotian upper secondary schools and provided the educational program to adolescent students. The recruitment of a control group allowed us to make external comparisons of the effect of the program on the experimental group. This study also has a substantial sample size—A specific and underrepresented target population in Southeast Asia. Our study design also included the delivery of the intervention in real-world settings. However, further studies on environmental health education are recommended to confirm our findings. A limitation of the study was that the Girls’ Integrated Health Care Program could not control for the effects of other education programs in parallel with the Laotian school curriculum. Moreover, as we assessed the intervention effects of our environmental health education immediately after the final session, we could not evaluate the effect’s retention for a longer period, such as one month, after the education. Nevertheless, our assessment of the short-term effect of our program can help future researchers to identify and develop educational programs that can have longer lasting effects. Finally, since our study developed the educational materials initially in English, some results could have been affected by translation bias [37]. To reduce any possible bias, the Laotian teachers developed the materials together with the researchers, and they were edited under the supervision of the Ministry of Education and Sports of Lao PDR.

## 5. Conclusions

In summary, we demonstrated that integrated health education improved the risk perception and information-seeking behavior regarding air pollution and chemical exposure of Laotian upper secondary school students. Our study highlights the importance of educational programs for Laotian students and suggests that our short-term health education improved the risk perception of adolescent girls more than that of adolescent boys. Further studies should establish stronger psychometric properties for questionnaires to measure such health-related behaviors. Moreover, larger-scale and longer-term randomized controlled trials are recommended to confirm our findings in the future.

## Figures and Tables

**Table 1:** Questionnaire measuring behaviors related to air pollution and chemical exposure.

Instrument	Question	Possible Range
Risk perception	1. How great a risk do you consider air pollution and chemical exposure to be to people and society?	1–7 ^a^
	2. How afraid are you when you think of air pollution and chemical exposure?	
	3. How harmful do you think air pollution and chemical exposure will be to the future development of society?	
	4. How worried are you about air pollution and chemical exposure?	
Self-efficacy	1. I have confidence in my ability to understand relevant information about air pollution and chemical exposure.	
	2. I have confidence in my ability to search for relevant information about air pollution and chemical exposure.	
	3. I have confidence in my ability to evaluate the reliability of relevant information about air pollution and chemical exposure.	
Information seeking	1. I intend to search for information about air pollution and chemical exposure.	
	2. I often focus on relevant information about air pollution and chemical exposure.	
	3. I often search for relevant information about air pollution and chemical exposure.	

^a^ Likert scale (1 = Not at all, 2 = Poorly, 3 = Slightly, 4 = Neutral, 5 = Moderately, 6 = Very, 7 = Extremely).

**Table 2:** Characteristics of study participants ^a.^

Variables	Total (*n* = 578)		Experimental Group (*n* = 370)	Control Group (*n* = 208)	*p*-Value ^b^
School grade	5th	261 (45.2)	180 (48.7)	81 (38.9)	0.02
	6th	317 (54.8)	190 (51.4)	127 (61.1)	
Age (years)		15.83 (0.96)	15.85 (0.98)	15.78 (0.92)	0.36
Sex	Boy	235 (40.7)	162 (43.8)	73 (35.1)	0.04
	Girl	343 (59.3)	208 (56.2)	135 (64.9)	
BMI ^c^	<18.5	64 (32)	31 (31)	33 (33)	0.88
	18.5–25	116 (58)	58 (58)	58 (58)	
	≥25	20 (10)	11 (11)	9 (9)	
Smoking	Never	551 (95.7)	350 (94.6)	201 (97.6)	0.02
	Ever	25 (4.3)	20 (5.4)	5 (2.4)	
Drinking	Never	273 (47.6)	188 (51.1)	85 (41.3)	0.09
	Ever	301 (52.4)	180 (48.9)	121 (58.7)	

^a^ Data are represented as number (%), mean ± SD. ^b^ Chi-square and *t*-tests were used to compare the demographic characteristics of the two groups. ^c^ BMI was only measured among the girl students.

**Table 3:** Summary statistics for ANCOVA comparing post-test scores after controlling for covariates ^a^.

	Pre-Test Mean (SD)	Post-Test Mean (SD)	Main Effect		
			F Statistic	*p*-Value	Partial eta Squared ($\eta_{p}^{2}$)
Risk perception			5.42	0.02	0.03
Control group	22.99 (3.19)	22.57 (3.25)			
Experimental group	22.61 (4.00)	23.38 (3.68)			
Self-efficacy			2.47	0.12	0.01
Control group	11.35 (3.42)	12.80 (3.24)			
Experimental group	12.02 (3.42)	13.87 (3.23)			
Information seeking			9.97	<0.01	0.05
Control group	11.82 (3.54)	12.74 (3.21)			
Experimental group	11.91 (3.64)	13.83 (3.42)			

^a^ Baseline pre-test scores, gender, school grade, drinking habits, and smoking habits were included as covariates in all analyses.

## Supplementary Materials

The following are available online at https://www.mdpi.com/1660-4601/17/14/5055/s1, Table S1: Cronbach’s alpha coefficient of the instruments, Table S2: Summary statistics for ANCOVA comparing post-test scores between girls after the different educational activities ^a^, Table S3: Comparison between pre-test and post-test scores stratified by smoking habits.
