# Supplementary material for: Impact of an Educational Program on Behavioral Changes toward Environmental Health among Laotian Students
Source: Int J Environ Res Public Health. 2020 Jul 14;17(14):5055. doi: 10.3390/ijerph17145055 (PMC7400243; doi:10.3390/ijerph17145055)
Supplement: Supplementary file 1 [file ijerph-17-05055-s001.pdf]

Table S1. Cronbach's alpha coefficient of the instruments.

Instrument	Cronbach's Alpha Coefficient
Risk perception pre-test	0.75
Risk perception post-test	0.82
Knowledge perceived self-efficacy pre-test	0.82
Knowledge perceived self-efficacy post-test	0.88
Information seeking pre-test	0.80
Information seeking post-test	0.85

Table S2. Summary statistics for ANCOVA comparing post-test scores between girls after the different educational activities [a].

	Pre-test Mean (SD)	Post-test Mean(SD)	Main Effect		
			**F Statistic**	***p*-Value**	**Partial eta Squared (η_p^2)**
Risk perception			0.39	0.53	0.00
Education only group [b]	23.24 (3.36)	24.02 (3.28)			
Education + special activity group [c]	22.83 (3.91)	24.12 (3.33)			
Self-efficacy			0.17	0.68	0.00
Education only group [b]	12.11 (2.97)	14.21 (3.11)			
Education + special activity group [c]	12.07 (3.02)	14.04 (3.11)			
Information seeking			0.02	0.89	0.00
Education only group [b]	12.30 (3.26)	14.5 (3.09)			
Education + special activity group [c]	12.13 (3.51)	14.42 (3.02)			

[a] Baseline pre-test scores, school grade, drinking habits, and smoking habits are included as covariates in all analyses. [b] Girls who participated in Girls' Integrated Health Care Education only. [c] Girls who participated in both Girls' Integrated Health Care Education and Peer Girls' Special Group Activity.

Table S3. Comparison between pre-test and post-test scores stratified by smoking habits.

	Instrument	*N*	Pre-test Mean (SD)	Post-test Mean (SD)	Sig. (2-tailed)
				**Experimental group**	
**Never Smoker [a]**	**Risk perception**	347	22.62(3.97)	23.41(3.67)	**<0.01**
	**Perceived self-efficacy**	347	12.10(3.41)	13.87(3.26)	**<0.01**
	**Information seeking**	347	11.97(3.59)	13.80(3.44)	**<0.01**
				**Control group**	
	**Risk perception**	199	22.98(3.19)	22.58(3.20)	0.13
	**Perceived self-efficacy**	199	11.41(3.40)	12.81(3.26)	**<0.01**
	**Information seeking**	199	11.80(3.67)	12.76(3.20)	**<0.01**
				**Experimental group**	
**Ever Smoker [b]**	**Risk perception**	20	20.35(3.70)	22.95(3.97)	0.59
	**Perceived self-efficacy**	20	10.65(3.27)	13.85(2.74)	**<0.01**
	**Information seeking**	20	10.85(4.18)	14.50(3.12)	**<0.01**
				**Control group**	
	**Risk perception**	5	23.00(3.54)	24.00(2.00)	0.63
	**Perceived self-efficacy**	5	10.00(1.87)	13.40(2.70)	0.06
	**Information seeking**	5	13.20(4.09)	14.00(2.35)	1.00

[a] Comparison between pre- and post-test scores was examined using paired-t test. [b] Comparison between pre- and post-test scores was examined using Wilcoxon signed-rank test.
